# Angiogenic Gene Therapy for Lower Extremity Ischemia: Experimental Advances and Clinical Experience

**DOI:** 10.3390/cells15121104

**Published:** 2026-06-18

**Authors:** Igor Samatoshenkov, Elena Zakirova, Julia Samatoshenkova, Albert Rizvanov, Yana Mukhamedshina

**Affiliations:** 1 Department of Vascular and Endovascular Surgery, Klinik Mittelbaden Rastatt-Forbach, 76437 Rastatt, Germany; 2 Institute of Fundamental Medicine and Biology, Kazan (Volga Region) Federal University, 420008 Kazan, Russia; lenahamzina@yandex.ru (E.Z.); rizvanov@gmail.com (A.R.); yana.k-z-n@mail.ru (Y.M.); 3 SAHI Republican Clinical Hospital of the Ministry of Health of the Republic of Tatarstan, 420138 Kazan, Russia; julia.samatoshenkova@mail.ru; 4 Division of Medical and Biological Sciences, Tatarstan Academy of Sciences, 420111 Kazan, Russia; 5 Department of Histology, Cytology and Embryology, Kazan State Medical University, 420012 Kazan, Russia

**Keywords:** chronic limb-threatening ischemia, peripheral arterial disease, therapeutic angiogenesis, gene therapy, viral vectors, non-viral vectors, plasmid DNA, adenoviral vectors, VEGF, FGF

## Abstract

Peripheral arterial disease and critical limb-threatening ischemia remain major clinical challenges, particularly in patients who are not candidates for surgical or endovascular revascularization. These limitations have stimulated extensive investigation into therapeutic angiogenesis using gene therapy approaches. This review summarizes experimental and clinical studies employing non-viral and viral gene delivery systems for the transfer of angiogenic factors, including VEGF, FGF-2, HGF, HIF-1α, and SDF-1. Particular attention is given to vector platforms, study design, patient populations, clinical endpoints, and safety outcomes reported in preclinical investigations and clinical trials. Although several studies demonstrated biological activity and favorable safety profiles, randomized trials such as RAVE and TAMARIS failed to demonstrate consistent and significant clinical efficacy. These findings emphasize the translational challenges associated with therapeutic angiogenesis and highlight the persistent gap between promising preclinical data and clinical outcomes in humans. Future progress in the field will likely depend on improved vector engineering, tissue-specific and regulated gene expression systems, optimized delivery strategies, and the integration of gene therapy with emerging regenerative technologies.

## 1. Introduction

Peripheral artery disease (PAD) represents one of the most prevalent vascular conditions worldwide, affecting approximately 230 million individuals, of whom roughly 11% present with chronic limb-threatening ischemia (CLTI) [1]. The disease is uncommon below the age of 50; however, in older age groups it affects up to 20% of the population regardless of sex [2]. Over recent years, the number of patients with lower limb peripheral artery disease has increased by 28.7% in low- and middle-income countries and by 13.1% in high-income countries [3]. The most significant risk factors include hyperlipidemia, hypertension, diabetes mellitus, chronic kidney disease, and smoking. The coexistence of three or more of these factors increases the risk of disease development tenfold [4]. The population prevalence and economic burden of lower limb ischemia are expected to continue rising, driven both by population ageing and by the adverse effects of smoking and diabetes on cardiovascular health [5].

Management of lower limb peripheral artery disease encompasses lifestyle modification, smoking cessation, and physical therapy, alongside secondary prevention pharmacotherapy including statins, antiplatelet agents, angiotensin-converting enzyme inhibitors, and angiotensin receptor blockers. Surgical intervention, specifically revascularization, should be considered in patients with lifestyle-limiting intermittent claudication or the presence of trophic tissue changes. Patients presenting with CLTI require immediate referral to a vascular surgeon [6].

Despite substantial advances in surgical and endovascular treatment modalities, revascularization outcomes remain highly variable and are largely contingent upon a complex interplay of clinical, anatomical, pathophysiological, and organisational factors. Restoration of macrovascular flow does not in itself guarantee limb preservation, as clinical outcomes are determined not solely by the patency of the reconstructed vessels but also by the functional state of the microcirculation, the regenerative capacity of the tissues, and the magnitude of the systemic inflammatory response [7].

The most favorable revascularization outcomes are observed in patients at earlier disease stages, when ischemia has not yet been complicated by extensive necrotic changes or infectious sequelae [8]. Preserved microcirculation and a well-developed collateral network facilitate effective flow redistribution following intervention, thereby promoting the regression of ischemic symptoms and the healing of trophic defects [9]. The anatomy of the vascular bed plays an equally important role: the patency of distal crural or pedal arteries and a limited longitudinal extent of occlusive lesions substantially enhance both the technical feasibility of revascularization and its long-term durability [10]. An additional favorable determinant is the preserved angiogenic and neurotrophic potential of the tissues, which supports capillary network restoration, improvement of innervation, and recovery of functional status in the ischemic limb [11]. Nevertheless, despite the availability of diverse surgical and endovascular treatment options, approximately 20–40% of patients may be ineligible for revascularization on medical or anatomical grounds [12].

The outcomes of lower limb ischemia treatment are thus governed by the balance between the possibilities afforded by macrovascular revascularization and the capacity of the tissues to respond to restored perfusion through microcirculatory adaptation and regeneration. The persistently high amputation rates observed in patients with CLTI underscore the limitations of conventional therapeutic approaches and highlight the imperative for developing adjunctive strategies targeting angiogenesis stimulation, microcirculatory improvement, and support of neuroprotective mechanisms [11,13].

Gene therapy is currently being considered as a strategy aimed at stimulating therapeutic angiogenesis, improving microcirculation, and supporting neurovascular mechanisms within ischemic tissues. However, despite promising results from preclinical investigations, the clinical efficacy of gene-based strategies in lower limb ischemia remains heterogeneous and is substantially dependent on the selection of therapeutic targets, delivery vectors, and disease stage. In recent years, considerable attention has been directed toward combinatorial regenerative approaches that integrate gene therapy with cell-based technologies, biomaterials, and tissue engineering platforms—strategies designed not only to augment the angiogenic response, but also to ensure more durable restoration of the microvasculature and neurovascular interactions. The present review systematically consolidates current evidence from experimental models and clinical investigations examining advanced gene therapy-based regenerative strategies in lower limb ischemia, and discusses the principal translational barriers, prospects for clinical implementation, and directions for future research.

## 2. Thrombo-Inflammatory and Metabolic Barriers to Therapeutic Angiogenesis in CLTI

Therapeutic angiogenesis in CLTI develops within a highly complex pathological microenvironment characterized not only by severe tissue hypoperfusion but also by chronic endothelial dysfunction, persistent inflammation, oxidative stress, metabolic dysregulation, platelet activation, and a sustained prothrombotic state. These interconnected mechanisms profoundly influence the endogenous angiogenic response and may substantially reduce the efficacy of exogenously administered angiogenic factors or gene-based therapeutic approaches. In advanced peripheral arterial disease, prolonged ischemia leads to progressive endothelial injury accompanied by impaired nitric oxide (NO) bioavailability, reduced endothelium-dependent vasodilation, increased vascular stiffness, and altered mechanotransduction signaling. Dysfunctional endothelial cells exhibit diminished responsiveness to vascular endothelial growth factor (VEGF), fibroblast growth factor (FGF), and hypoxia-inducible signaling pathways, thereby limiting adaptive neovascularization [8,10].

Chronic inflammation represents another critical determinant of vascular dysfunction in CLTI. Atherosclerotic plaques and ischemic tissues are infiltrated by activated macrophages, neutrophils, and T lymphocytes that release tumor necrosis factor-α (TNF-α), interleukin-1β, interleukin-6, interferon-γ, and matrix metalloproteinases (MMPs) [14]. Although transient inflammatory signaling may initially promote angiogenesis through cytokine-mediated endothelial activation, persistent inflammation ultimately contributes to extracellular matrix degradation, endothelial apoptosis, capillary rarefaction, and impaired vessel maturation. Excessive proteolytic activity may additionally degrade endogenous and exogenous growth factors, limiting their biological availability within ischemic tissues.

The thrombo-inflammatory component of CLTI further complicates therapeutic vascular regeneration. Platelet activation and thrombin generation are markedly enhanced in advanced peripheral arterial disease, particularly in diabetic patients [15]. Activated platelets release VEGF, platelet-derived growth factor (PDGF), transforming growth factor-β (TGF-β), and inflammatory mediators that may transiently support angiogenesis; however, chronic platelet activation also promotes leukocyte recruitment, endothelial dysfunction, oxidative stress, and microvascular thrombosis. Thrombin signaling through protease-activated receptors (PARs) contributes to endothelial activation and vascular remodeling but may simultaneously exacerbate tissue injury and impair microcirculatory perfusion [16]. In addition, fibrin deposition and microthrombus formation within the distal microvasculature can create a structural barrier to effective tissue reperfusion despite successful induction of capillary sprouting. Importantly, induction of neovessel formation does not necessarily translate into restoration of functional tissue perfusion. Newly formed vessels generated under ischemic and inflammatory conditions are frequently structurally immature, poorly organized, hyperpermeable, and characterized by insufficient pericyte and smooth muscle cell coverage [17]. Inadequate vessel stabilization may lead to regression of neocapillaries and failure to establish durable collateral circulation. Effective therapeutic angiogenesis therefore requires coordinated regulation not only of endothelial proliferation and migration but also of arteriogenesis, extracellular matrix remodeling, mural cell recruitment, and long-term vascular maturation.

Emerging evidence suggests that impaired angiogenic responsiveness in CLTI reflects a multifactorial failure of the entire neurovascular and regenerative niche rather than isolated deficiency of single growth factors. Skeletal muscle degeneration, peripheral neuropathy, mitochondrial dysfunction, stem cell exhaustion, and senescence-associated signaling collectively contribute to defective tissue regeneration and impaired adaptive vascular remodeling [18]. These observations may partly explain the discrepancy between promising preclinical results and the relatively modest outcomes observed in clinical trials of angiogenic growth factor and gene therapies. Consequently, future therapeutic strategies for CLTI may require integrated multimodal approaches targeting angiogenesis together with endothelial restoration, immunomodulation, antithrombotic therapy, oxidative stress reduction, and metabolic correction.

## 3. Platelets and Therapeutic Angiogenesis in Ischemic Tissue

Platelets are increasingly recognized as active regulators of vascular regeneration and tissue remodeling rather than merely mediators of hemostasis. Beyond their classical role in thrombosis, platelets represent a major reservoir of angiogenic and immunomodulatory mediators that critically influence ischemic tissue repair. Activated platelets release numerous growth factors and cytokines from α-granules, including VEGF, PDGF, TGF-β, angiopoietin-1 (ANGPT1), FGF, and epidermal growth factor (EGF), all of which participate in endothelial activation, extracellular matrix remodeling, and neovessel maturation [19]. Platelet-derived VEGF is a potent stimulator of endothelial proliferation, migration, and capillary sprouting, whereas PDGF contributes predominantly to pericyte recruitment, stabilization of nascent vessels, and fibroblast activation. TGF-β further modulates inflammatory resolution, extracellular matrix deposition, and macrophage polarization toward reparative phenotypes. Importantly, platelets may also release anti-angiogenic mediators depending on the activation context and local microenvironment, indicating that platelet signaling exerts bidirectional regulatory effects on angiogenesis [19].

In CLTI, persistent endothelial dysfunction, hypoxia, inflammation, and microvascular rarefaction are accompanied by enhanced platelet activation and platelet–leukocyte interactions. Platelets contribute to inflammatory cell recruitment, endothelial permeability, and remodeling of the ischemic microcirculation. At the same time, platelet-derived growth factors may partially compensate for impaired endogenous regenerative signaling by supporting angiogenesis and granulation tissue formation [20]. These mechanisms may be particularly relevant in the context of therapeutic angiogenesis and gene therapy approaches utilizing *VEGF*-expressing constructs. *VEGF* overexpression can potentially interact with platelet activation pathways, as VEGF itself modulates endothelial permeability and platelet-endothelial crosstalk. Moreover, synergistic signaling between VEGF and PDGF pathways appears essential for the formation of stable and functional vasculature, since VEGF-induced endothelial sprouting alone may generate immature and hyperpermeable vessels in the absence of adequate mural cell recruitment. Experimental studies have demonstrated that combined VEGF/PDGF signaling improves vascular maturation and tissue repair more effectively than isolated VEGF stimulation [21].

Another clinically important consideration is the influence of background antiplatelet therapy on therapeutic angiogenesis. Since dual antiplatelet or aspirin-based therapy constitutes standard treatment in patients with peripheral artery disease and CLTI, modulation of platelet activation may alter the local availability of platelet-derived angiogenic mediators. Although suppression of excessive platelet activation may reduce thrombo-inflammatory injury and improve microvascular perfusion, it may simultaneously attenuate the release of regenerative growth factors involved in tissue repair. The overall impact of antiplatelet therapy on angiogenic efficacy therefore remains incompletely understood and warrants further investigation in future translational and clinical studies [19].

## 4. Diabetes-Associated Impairment of Therapeutic Angiogenesis in CLTI

Diabetes mellitus represents one of the most important factors contributing to the progression of CLTI, and diabetic patients constitute the largest subgroup of “no-option” patients considered for experimental angiogenic therapies. However, despite extensive investigation of therapeutic angiogenesis in diabetic ischemic disease, clinical efficacy remains limited. Accumulating evidence suggests that the diabetic microenvironment profoundly impairs endogenous vascular repair mechanisms and significantly reduces responsiveness to angiogenic gene therapy.

A central role in the impaired angiogenic response observed in CLTI is played by diabetes mellitus and associated metabolic disturbances. Chronic hyperglycemia induces mitochondrial dysfunction and excessive production of reactive oxygen species (ROS), which contribute to oxidative damage of endothelial cells and circulating endothelial progenitor cells (EPCs). In diabetic tissues, advanced glycation end products (AGEs) accumulate within the extracellular matrix and vascular wall, activating the receptor for AGEs (RAGE) and promoting NF-κB-mediated inflammatory signaling [22]. This process enhances the expression of proinflammatory cytokines, adhesion molecules, and tissue factor, thereby sustaining endothelial activation and vascular inflammation. Simultaneously, oxidative stress destabilizes hypoxia-inducible factor-1α (HIF-1α), resulting in impaired transcriptional activation of VEGF and other hypoxia-responsive genes despite persistent ischemia [23]. Consequently, diabetic ischemic tissues frequently demonstrate a paradoxical coexistence of severe hypoxia and insufficient angiogenic compensation.

Importantly, the diabetic ischemic microenvironment is characterized not only by macrovascular occlusion but also by severe microvascular dysfunction, chronic inflammation, neuropathy, and tissue fibrosis. Capillary basement membrane thickening, pericyte dysfunction, and impaired skeletal muscle regeneration further compromise tissue oxygenation and vascular responsiveness [24]. As a result, stimulation of angiogenesis alone may be insufficient to restore tissue integrity in advanced diabetic CLTI. These pathophysiological abnormalities likely contribute to the limited efficacy of first-generation angiogenic vectors in diabetic patients. Non-viral plasmid systems often exhibit poor transfection efficiency and transient gene expression in chronically inflamed and fibrotic diabetic tissues. Viral vectors may achieve higher transgene expression but remain affected by impaired cellular responsiveness to angiogenic signaling and by persistent inflammatory activation. In addition, severe microvascular rarefaction may limit adequate vector distribution within ischemic tissues.

Several clinical studies specifically targeting diabetic populations have demonstrated both the therapeutic potential and the limitations of angiogenic gene therapy. VM202, a plasmid encoding hepatocyte growth factor (*HGF*), demonstrated promising results in painful diabetic neuropathy, suggesting potential neurovascular regenerative effects [25]. Similarly, Barć et al. reported encouraging findings using combined *VEGF/HGF* gene therapy in diabetic patients with critical limb ischemia [26]. Nevertheless, larger randomized studies are still required to establish durable clinical efficacy and identify patient subgroups most likely to benefit from such approaches.

Given the complex pathophysiology of diabetic ischemic disease, future therapeutic strategies will likely require multimodal approaches combining angiogenic stimulation with immunomodulation, metabolic correction, neurovascular repair, and tissue regeneration. Emerging approaches involving multicistronic vectors, exosome-mediated delivery, biomaterial-assisted platforms, and combined gene-cell therapies may help overcome some of the biological barriers associated with the diabetic microenvironment.

## 5. Key Historical Landmarks in Evolution of Gene Therapy in Lower Extremity Ischemia

**Figure 1:**
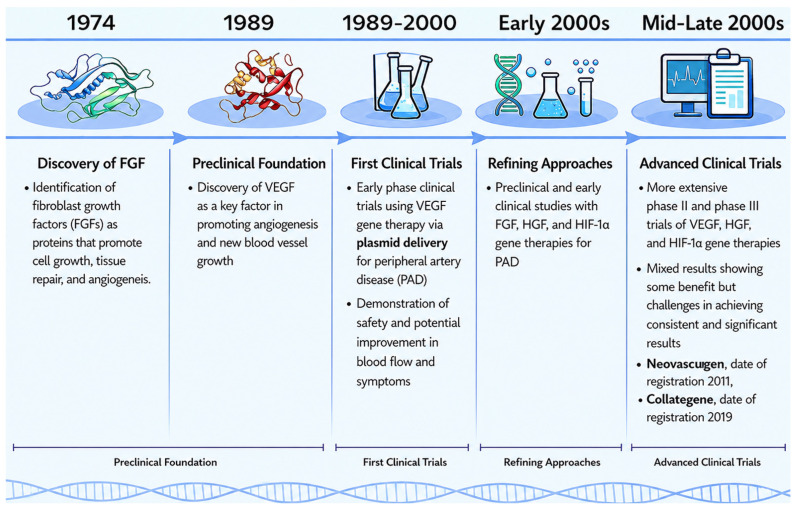
Historical timeline of gene therapy development in lower extremity ischemia.

The development of gene therapy for lower extremity ischemia has progressed through a series of pivotal experimental and clinical milestones, reflecting the gradual transition from proof-of-concept studies to translational and early clinical applications, as summarized in Figure 1. The formation of the modern concept of angiogenesis as a strictly regulated biological process began in the 1970s. Judah Folkman’s 1971 work played a key role, who formulated the hypothesis that tumor growth critically depends on the induction of neoangiogenesis and proposed the existence of specific factors that stimulate vascular growth [27]. This concept laid the foundation for the search for molecular regulators of vascular growth.

Among the first identified angiogenic molecules were members of the FGF family. In 1974, Denis Gospodarowicz isolated a mitogenic factor from bovine pituitary extracts that exhibited broad proliferative activity across multiple cell types, including fibroblasts. This factor was later identified as basic fibroblast growth factor (bFGF/FGF-2). It should be noted that the terminology FGF-1 and FGF-2 was introduced only subsequently, following molecular cloning studies such as those by David Abraham et al. (1986), which established the distinction between acidic (FGF-1) and basic (FGF-2) fibroblast growth factors [28]. Importantly, the angiogenic activity of FGF in vivo had already been demonstrated by Gospodarowicz et al. in 1979 [29], whereas later studies, including those by Peter Böhlen et al. (1984), focused on its biochemical and molecular characterization [30].

A decisive stage in the development of the molecular biology of angiogenesis was the discovery of vascular endothelial growth factor (VEGF). In 1986, Senger and colleagues described a vascular permeability factor (VPF) secreted by tumor cells [31]. In 1989, Napoleone Ferrara and colleagues independently isolated VEGF from bovine pituitary follicular stellate cells, demonstrating that it is a specific mitogen for endothelial cells and introducing the term VEGF (Ferrara coined the term VEGF, and the designation VEGF-A came into use following the discovery of VEGF-B, -C, and -D to distinguish between members of the family) [32]. Subsequent molecular cloning of the corresponding gene was achieved almost simultaneously by several groups in 1989, including Leung et al. and Keck et al., demonstrating that VEGF and vascular permeability factor (VPF) are encoded by the same gene [33,34]. Around the same time, Jean Plouët and colleagues independently purified a similar endothelial mitogen from AtT 20 pituitary cells, which they initially named vasculotropin [35]. In the early 1990s, the principal VEGF receptors—VEGFR-1, VEGFR-2, and VEGFR-3—were identified, laying the foundation for subsequent research into VEGF signaling [36,37,38].

In 1991, placental growth factor (PlGF), a member of the VEGF family that plays a role in pathological angiogenesis, was discovered [39]. Later, VEGF-C and VEGF-D were identified, expanding the understanding of the VEGF family by demonstrating its involvement not only in angiogenesis but also in lymphangiogenesis [40].

Studies of factors responsible for maturation and stabilization of the vascular wall developed concurrently, in the 1990s. It was shown that platelet-derived growth factor B (PDGF-B) plays a key role in pericyte recruitment and the formation of functionally mature vessels [41,42]. The endothelial-specific receptor tyrosine kinase Tie2 (also known as TEK) was identified and cloned during systematic searches for novel receptor tyrosine kinases, with one of the first descriptions reported by Runting et al. in 1993 [43]. Together with the closely related Tie1 receptor, it defined a new subclass of endothelial-enriched receptors implicated in vascular development. While its ligand remained initially unknown, this was resolved with the discovery of ANGPT in the mid-1990s, particularly ANGPT1 as a Tie2 agonist [44,45].

Thus, by the end of the 1990s, a multilevel model of angiogenesis had been formed, including VEGF, regulators of permeability and remodeling, and molecules ensuring maturation and functional stability of newly formed vessels (PDGF-B, ANGPT1/2). These discoveries laid the foundation for the development of therapeutic angiogenesis strategies and gene therapy for ischemic diseases.

The transition from fundamental molecular discoveries in angiogenesis (1970s–1990s) to clinical application is often presented too abruptly, overlooking a crucial intermediate stage—preclinical validation in animal models. In reality, the translation of angiogenic factors such as VEGF and FGF into therapeutic strategies was critically enabled by the development of reproducible in vivo models of ischemia. A landmark advance was the development of the hindlimb ischemia model by Ryuichi Takeshita et al. in 1994, which allowed quantitative assessment of neovascularization, perfusion recovery, and functional outcomes following administration of angiogenic factors [46]. Subsequently, this and related models (including rabbit and murine systems) became indispensable platforms for testing protein delivery as well as emerging gene therapy approaches, demonstrating proof-of-concept and establishing dose–response relationships. Subsequent studies by Jeffrey M. Isner and colleagues further advanced the field by applying *VEGF* gene transfer in ischemic tissues, helping to translate preclinical findings into early clinical investigation [47].

Gene therapy for lower extremity ischemia emerged in the mid-1990s within the concept of therapeutic angiogenesis, with early pilot and Phase I studies, particularly those by Jeffrey M. Isner and colleagues, demonstrating that local delivery of a plasmid encoding *VEGF*165 could induce neoangiogenesis and improve perfusion in patients with CLTI, thereby supporting further development of this approach [48,49]. In the 2000s, with the initiation of large-scale clinical trials, the spectrum of therapeutic genes was expanded to include FGF, HGF, and hypoxia-inducible factor 1α (HIF-1α), and various delivery systems were developed, including plasmid and viral vectors.

Several gene therapy products have been developed for therapeutic angiogenesis in peripheral artery disease. Notably, Neovasculgen (a plasmid encoding human *VEGF*165, date of registration 2011) and Collategene (a plasmid encoding *HGF*, date of registration 2019) have undergone clinical evaluation.

In recent years, researchers’ attention has shifted toward combined gene therapy strategies, including the simultaneous expression of angiogenic and cytoprotective factors, or multiple complementary angiogenic factors, as well as optimization of dosing and routes of administration. In particular, studies involving administration of gene combinations in recent years have shown promising results in patients with lower extremity ischemia, including those with diabetes mellitus, underscoring the potential of a multifactorial approach in the treatment of lower limb ischemia; these studies are discussed below [26].

Taken together, the above historical milestones illustrate how early gene therapy strategies for lower extremity ischemia evolved from exploratory angiogenesis concepts to more advanced approaches based on pathogenetic mechanisms. However, it should be taken into account that the therapeutic efficacy of gene constructs extends beyond the mere induction of neovascularization. Fundamental knowledge of the complex interactions among angiogenic, cytoprotective, and tissue-stabilizing mechanisms is of great importance for understanding the therapeutic potential, limitations, and context-dependent outcomes. In this context, the following section provides a comprehensive overview of the biological effects of key angiogenic and protective factors that can be leveraged in gene therapy-based strategies for the treatment of lower extremity ischemia.

## 6. Key Angiogenic and Protective Factors VEGF

Among angiogenic mediators, VEGF remains the most extensively studied protein in both experimental models and clinical settings. VEGF constitutes a family of heterogeneous ligands (VEGF-A, VEGF-B, VEGF-C, VEGF-D, and PlGF) that interact with tyrosine kinase receptors VEGFR1 (Flt1), VEGFR2 (KDR/Flk1), and VEGFR3 (Flt4), as well as with co-receptors neuropilin-1/2 and membrane proteoglycans such as Syndecan-2, which modulate ligand affinity, receptor homo- and heterodimerization, and the spatiotemporal organization of signaling [50,51].

VEGF-A generates multiple isoforms as a result of alternative mRNA splicing, which differ in length and in their capacity to bind the extracellular matrix (ECM) and co-receptors. The principal isoforms include VEGF121, VEGF165, VEGF189, and VEGF206, which vary in their heparin-binding properties, thereby determining their distribution and bioavailability. VEGF165 is the most abundant and biologically active isoform, whereas VEGF121 also exhibits substantial angiogenic activity. VEGF189 displays a more localized function due to its strong association with the extracellular matrix. This diversity of isoforms provides flexible spatiotemporal regulation of angiogenesis under both physiological and pathological conditions, including vascular growth, remodeling, and tumor progression [52].

VEGF-A is a key mediator of angiogenesis and vascular permeability. VEGFR2 serves as the principal signaling receptor for VEGF-A; upon activation, it undergoes dimerization, trans-autophosphorylation of the kinase domain (critical residues Y1054/Y1059), and phosphorylation of multiple tyrosine residues within the C-terminal region, including Y1175 and Y1214. These events enable recruitment of phospholipase C-γ (PLCγ), Ras–MAPK cascade adaptors, and components of the endothelial mechanosensitive response to blood flow. Activation of VEGFR2 initiates three major signaling pathways: (1) the PI3K–Akt pathway promotes endothelial cell survival, activates eNOS, and stimulates nitric oxide production, leading to vasodilation and endothelial protection; (2) the Ras–Raf–MEK–ERK pathway regulates cell proliferation, G1/S phase transition, and the expression of cyclins and growth factors; (3) the PLCγ–PKC–Ca^2+^ pathway induces PIP2 hydrolysis with generation of IP3 and DAG, elevation of intracellular Ca^2+^ levels, activation of PKC, and rapid remodeling of intercellular junctions. The duration and localization of these signals depend on intracellular trafficking of VEGFR2, including endosomal MAPK signaling [53]. VEGF signaling via VEGFR2 is further regulated by the Notch pathway, which ensures proper vascular patterning, whereas disruption of VEGF–Notch balance results in a disorganized and excessively branched vascular network [50].

VEGF-B (isoforms B167/B186) predominantly binds to VEGFR1 and neuropilin-1 without activating VEGFR2, supporting endothelial cell survival in the heart, promoting coronary angiogenesis, conferring protection under ischemic conditions, and regulating lipid metabolism in adipocytes. In contrast to VEGF-A, VEGF-B does not increase vascular permeability and only weakly contributes to tumor angiogenesis. VEGF-C and VEGF-D primarily activate VEGFR3 and mediate lymphangiogenesis, with possible engagement of VEGFR2 at high ligand concentrations, leading to a combined angio- and lymphangiogenic response [54].

Placental growth factor (PlGF) comprises four isoforms (PlGF- 1–4), predominantly binds to VEGFR1 (Flt1) and neuropilin-1, and does not activate VEGFR2. PlGF stimulates endothelial cell proliferation and migration under non-ischemic conditions, contributes to placental vascular remodeling, and promotes angiogenesis in embryonic organs through synergistic interaction with VEGF-A via VEGFR1/2 heterodimers. Physiologically, PlGF levels increase from the 11th week of gestation (peaking around week 30) and decline toward term [50].

At the physiological level, VEGF is critical for embryonic vasculogenesis—heterozygous loss of VEGF in experimental models results in embryonic lethality [50]. In the adult organism, VEGF fulfills multiple functions, including wound healing [55], coronary and collateral vessel growth in ischemia [56], maintenance of endothelial homeostasis [57], and preservation of pericyte coverage of capillaries [58]. Beyond its vascular effects, VEGF exerts a broad spectrum of extravascular functions. In the central nervous system, VEGF-A acts as a neuroprotective factor, reducing the extent of ischemic injury in stroke models and improving outcomes through a combination of angiogenic effects (enhanced perfusion) and direct actions on neurons and glial cells. In neurodegenerative diseases, particularly Alzheimer’s disease, increased VEGF levels have been associated with better hippocampal preservation, reduced cognitive decline, and enhanced microglial phagocytosis of amyloid-β [59,60,61]. Within the immune system, VEGF-A, primarily via VEGFR1 on monocytes/macrophages and endothelial cells, promotes their recruitment to sites of inflammation, modulates adhesion molecule expression and cytokine networks, and simultaneously contributes to inflammatory vascular damage and edema while participating in endothelial repair and regeneration [50,62].

It should be emphasized that VEGF production is not constant but dynamically regulated in accordance with the organism’s physiological demands. A clinical study demonstrated that chronic ischemia is associated with a pronounced local increase in VEGF levels in skeletal muscle. Prior to revascularization, VEGF concentrations in ischemic calf muscle (m. gastrocnemius) were significantly higher than in non-ischemic control muscle (m. vastus lateralis), reflecting activation of a hypoxia-induced angiogenic response. Following successful restoration of blood flow, VEGF levels in the previously ischemic muscle markedly decreased and approached those of control tissue, whereas no significant changes were observed in non-ischemic muscle. These findings indicate that VEGF serves as a sensitive marker of local tissue hypoxia in limb ischemia and reflects an adaptive, yet insufficiently effective, endogenous attempt to stimulate angiogenesis, which does not prevent progression of ischemia in the absence of interventions aimed at restoring perfusion [63].

**FGF**

FGF is a large family of heparin-binding growth factors comprising at least 20 structurally related proteins, among which FGF-1 and FGF-2 are classical angiogenic ligands. They interact with the family of tyrosine kinase receptors FGFR1–FGFR4 expressed on endothelial cells, smooth muscle cells, and fibroblasts, and activate intracellular signaling cascades (PI3K/Akt, MAPK/ERK, PLCγ) that stimulate proliferation, migration, and survival of vascular wall cells, forming the basis for new vessel formation. These factors play essential roles in physiological vascular development and wound healing, as well as in pathological processes of neoangiogenesis in tumors and chronic inflammation [64].

Classical FGFs do not always act as direct initiators of angiogenesis, but may regulate the expression of other key factors, most notably by stimulating VEGF-A and its receptors, which are critical for vascular permeability and the growth of new capillaries. In FGFR1-deficient models, disruption of FGF signaling reduces VEGF production and leads to defects in the vascular network, indicating a central role of FGF as a coordinator of other angiogenic systems in the process of neovascularization [65]. FGF-2 enhances the expression of pro-angiogenic proteins, including HGF and PDGF, and also stimulates the secretion of metalloproteinases involved in extracellular matrix remodeling, which is required for endothelial cell migration and vascular tube formation. In certain contexts, FGF signaling is required to maintain endothelial cell responsiveness to VEGF stimuli and to stabilize newly formed vessels [65]. Experimental data show that FGF-1 and FGF-2 are potent mitogens for endothelial cells in vitro and are capable of stimulating the formation of capillary-like structures, and their expression correlates with angiogenic activity during tissue injury and tumor growth. In contrast to VEGF, which acts on highly specialized endothelial receptors, the FGF ligand-receptor system is more universal in its cellular targeting, affecting multiple cell types of the vascular wall and stroma, which makes it an important mechanism of adaptive vascular plasticity [65].

It has been shown that in patients with chronic lower limb ischemia, the ischemic skeletal muscle exhibits pronounced structural and molecular signs of adaptation to prolonged hypoperfusion. In particular, capillaries in ischemic muscle acquire features of arteriolization, including thickening of the vascular wall and the appearance of smooth muscle cells, which is accompanied by a significant increase in *FGF*-2 expression in the endothelium and perivascular cells. The level of FGF-2 correlates with the severity of ischemia and the degree of vascular remodeling, indicating its key role in the induction of structurally more mature and stable vessels in chronic ischemic lesions. These findings indicate that FGF-2 is involved not only in capillary angiogenesis but also in the processes of arteriogenesis and vascular stabilization, which distinguishes it from the VEGF-mediated angiogenic response and underscores the potential of FGF-2 as a component of combined therapeutic strategies for chronic lower limb ischemia [66].

**HGF**

HGF is a pleiotropic mesenchymal cytokine that is predominantly secreted by stromal cells and acts on epithelial and endothelial cells via the specific tyrosine kinase receptor c-Met (MET). HGF regulates cell growth, migration, morphogenesis, and survival, making it a key factor in embryonic organ development, adult tissue regeneration, and wound healing. At the molecular level, HGF is produced as an inactive precursor (pro-HGF), which is activated by a serine protease at sites of tissue injury. The active form subsequently binds to c-Met on the surface of target cells, inducing receptor dimerization and activating tyrosine kinase signaling that promotes endothelial cell proliferation, migration, and survival (resistance to apoptosis) [67].

In vitro studies demonstrate that HGF induces endothelial cell growth and morphogenesis via ERK-, STAT3-, and AKT-dependent pathways, while also protecting these cells from hypoxia-induced apoptosis, thereby facilitating the formation of vascular structures in response to tissue injury or ischemia [68]. The molecular mechanisms of HGF action include upregulation of key angiogenic genes, such as VEGF, through activation of the transcription factor ETS-1 and associated c-Met → PI3K/Akt/ERK signaling pathways. This enhances endothelial cell migration and extracellular matrix remodeling, both of which are essential for neovascularization [69]. Thus, the release of HGF into the extracellular matrix increases VEGF activity during neoangiogenesis [70]. HGF also augments VEGF-A production in various cell types via a mechanism dependent on activation of HIF-1α, further amplifying the angiogenic response under hypoxic conditions [71].

Clinical studies have shown that patients with limb ischemia exhibit elevated serum levels of HGF compared to controls, whereas HGF concentrations decrease following revascularization, indicating its activation in response to tissue hypoxia and its involvement in adaptive vascular remodeling processes [72]. However, it is important to note the context-dependent effects of HGF on muscle regeneration, particularly when its activation is temporally dysregulated. A study by Miller et al. demonstrated that HGF plays a critical role in the early phase of skeletal muscle regeneration by activating quiescent satellite cells and stimulating their proliferation. Nevertheless, despite an increased number of myoblasts, HGF administration did not accelerate muscle tissue regeneration. On the contrary, HGF was shown to inhibit myoblast differentiation and myotube formation, maintaining cells in a proliferative state. These findings indicate that HGF primarily functions as an early activation signal for satellite cells, whereas subsequent muscle cell differentiation requires a reduction in its activity [73]. These properties have provided a rationale for the development of gene therapy approaches involving the delivery of plasmids encoding *HGF* (including in combination with *VEGF*) at different time intervals. Based on the outcomes observed in clinical studies, such strategies may, in the near future, become an effective treatment option for patients with CLTI [74].

**SDF-1**

Stromal cell-derived factor 1 (SDF-1) is a chemokine actively secreted by stromal cells (fibroblasts, mesenchymal stromal cells, and others) and plays a key role in the regulation of cell migration, survival, proliferation, and differentiation, and is an important mediator of angiogenesis and vasculogenesis [75]. SDF-1 functions predominantly through its receptors CXCR4 and CXCR7 on endothelial cells, endothelial progenitor cells (EPCs), smooth muscle cells, and other cell types [76], activating several intracellular cascades, including PI3K/Akt, MAPK/ERK, and NF-κB, which promotes enhanced survival, migration, and formation of vascular structures [77].

In the context of angiogenesis, SDF-1/CXCR4 signaling stimulates the proliferation and migration of endothelial cells and endothelial progenitor cells, enhances the formation of capillary-like structures in vitro, and increases vascular density in ischemia models in vivo. In particular, SDF-1 increases Akt and ERK activity, which is important for cellular survival and growth, whereas blockade of CXCR4 significantly suppresses these angiogenic effects [77]. In addition, different isoforms of CXCL12 (for example, CXCL12α and CXCL12β) exhibit differential efficacy in stimulating proliferation, preventing apoptosis, and promoting vascular network formation, reflecting the complex regulation of angiogenic processes under physiological and pathological conditions [78]. SDF-1 also acts as a potent chemotactic factor for endothelial progenitor cells and hematopoietic stem/progenitor cells, facilitating their mobilization from the bone marrow and recruitment to sites of ischemia or injury where neovascularization is required. This interaction is particularly important in the setting of limb ischemia, as local upregulation of SDF-1 expression creates a gradient that attracts CXCR4-positive cells to areas of neovascularization [79].

In addition to its direct effects on the endothelium, SDF-1 enhances the synthesis of other angiogenic factors, including VEGF, and participates in cross-signaling interactions with other mediators of angiogenesis, thereby forming a regulatory network that promotes vascular growth and stabilization of newly formed vessels [79]. Thus, the SDF-1/CXCR4 axis is considered a promising target for therapeutic angiogenesis in the treatment of ischemic diseases, as well as an important component of tissue regeneration mechanisms and vascular network reconstruction in response to injury [80].

It has been established that in patients with critical limb ischemia, activation of the SDF-1/CXCL12–CXCR4 axis occurs as an important component of the ischemia-induced reparative response. It has been shown that the expression of SDF-1α (CXCL12) is significantly increased in ischemic skeletal muscle compared with non-ischemic tissue, while the CXCR4 receptor is detected on microvascular endothelial cells and circulating endothelial progenitor cells. Induction of SDF-1 under ischemic conditions is mediated by HIF-1α-dependent mechanisms and promotes chemoattraction of CXCR4^+^ progenitor cells from the bone marrow to the hypoxic zone, thereby enhancing angiogenesis and neovascularization. However, despite increased local *SDF-1* expression, endogenous activation of this axis in patients with critical limb ischemia is insufficient to restore adequate perfusion, which underscores the limited capacity of spontaneous regenerative mechanisms and substantiates interest in therapeutic modulation of SDF-1/CXCR4 signaling in chronic lower limb ischemia [78].

**Angiopoietin**

ANGPT constitutes a family of vascular growth factors that play a key role in the maturation, stabilization, and remodeling of the vascular network, acting predominantly at the later stages of angiogenesis. The most extensively studied members are ANGPT1 and ANGPT2, which serve as ligands for the tyrosine kinase receptor Tie2, expressed primarily on endothelial cells. ANGPT1, produced by pericytes, smooth muscle cells, and stromal elements, functions as a Tie2 agonist and promotes vascular stabilization, strengthening of interendothelial junctions, reduction in vascular permeability, and enhanced endothelial survival via activation of the PI3K/Akt and eNOS signaling pathways [81].

In contrast, ANGPT2 is predominantly expressed by endothelial cells themselves and acts as a context-dependent antagonist of ANGPT1 or a partial agonist of Tie2, leading to destabilization of the vascular wall. ANGPT2 weakens endothelial interactions with pericytes and the extracellular matrix, increases vascular permeability, and thereby “primes” vessels for remodeling. In the presence of VEGF, this promotes active angiogenesis, whereas in the absence of VEGF it leads to vessel regression, underscoring the coordinating role of ANGPT2 in vascular dynamics [81]. Studies have also demonstrated that ANGPT2 can directly stimulate endothelial cell migration and sprouting independently of Tie2 by binding to integrins and activating migration-associated pathways (e.g., FAK/Rac1) in cells with low Tie2 expression, adding an additional level of regulation to the complex process of angiogenesis [82].

In angiogenesis, angiopoietins are not primary endothelial mitogens, unlike VEGF or FGF, but instead function as regulators of vascular stability and plasticity, determining whether vessels are maintained, remodeled, or undergo regression. Experimental models of skeletal muscle ischemia with the formation of ischemic ulcers have shown that combined activation of VEGF-dependent vascular growth and ANGPT1-mediated stabilization leads to the formation of a functionally mature and stable capillary network, whereas isolated VEGF stimulation is often associated with the development of immature and hyperpermeable vessels [81].

From a clinical and biological perspective, the ANGPT/Tie2 system exerts functionally opposing effects on vascular homeostasis. ANGPT1 primarily acts as a protective and vessel-stabilizing factor by activating Tie2 signaling, thereby promoting endothelial survival, strengthening endothelial-pericyte interactions, reducing vascular permeability, and supporting maturation of newly formed vessels. These properties make ANGPT1 a promising mediator of therapeutic angiogenesis in ischemic diseases. In contrast, ANGPT2 is typically upregulated under conditions of hypoxia, inflammation, and endothelial activation, where it antagonizes or destabilizes ANGPT1/Tie2 signaling. Increased ANGPT2 expression is associated with endothelial dysfunction, enhanced vascular permeability, inflammatory cell recruitment, and pathological vascular remodeling, particularly in tumors and chronic inflammatory diseases. Consequently, elevated ANGPT2 levels have been correlated with poor prognosis in both oncological and vascular disorders, making ANGPT2 an important target for anti-angiogenic and vascular-normalizing therapies [83].

In patients with chronic and critical limb ischemia, dysregulation of the ANGPT/Tie2 system has been demonstrated, characterized by increased ANGPT2 expression in ischemic skeletal muscle and microvascular endothelium in the context of relative ANGPT1 deficiency. This imbalance is associated with the formation of functionally immature and unstable vessels [84]. These findings highlight the important role of angiopoietins in coordinating the transition from vascular destabilization to maturation and underscore the therapeutic potential of ANGPT1-based strategies or combination strategies to enhance the efficacy of angiogenesis in CLTI.

**Angiogenin**

Angiogenin (ANG) is an angiogenic protein belonging to the ribonuclease family, first identified by its ability to induce the formation of new blood vessels in various biological models. ANG possesses weak ribonuclease activity, which has been shown to be critical for its biological function, distinguishing it from classical growth factors such as VEGF or FGF [85]. ANG activity is regulated by intracellular inhibitors, most notably the ribonuclease inhibitor (RI), binding to which suppresses its function [86]. The mechanisms of ANG action in angiogenesis involve activation of endothelial cells, stimulation of their migration, proliferation, and invasion, followed by the formation of tubular structures. ANG is capable of translocating into the nucleus of endothelial cells, where it enhances ribosomal RNA transcription, thereby promoting ribosome biogenesis, protein synthesis, and cell growth—processes required to sustain proliferation under the influence of other angiogenic factors. In addition, the interaction of ANG with cell surface actin on endothelial cells modifies cytoskeletal organization and facilitates extracellular matrix remodeling, thereby promoting angiogenic sprouting [87,88,89]. Beyond initiating neovascularization, ANG can also potentiate the effects of other angiogenic factors, such as VEGF and FGF-2, contributing to the formation of functionally mature vessels.

It has been established that patients with CLTI exhibit elevated levels of ANG in both plasma and ischemic muscle tissue, reflecting activation of endogenous angiogenic mechanisms in response to hypoxia. Nevertheless, clinical studies indicate that endogenous upregulation of ANG is insufficient to restore adequate perfusion in limb ischemia. This underscores the therapeutic potential of angiogenin modulation, for example through recombinant proteins or gene therapy, as part of combined strategies to stimulate angiogenesis in patients with CLTI [90].

**HIF 1α**

Hypoxia-inducible factor 1 alpha (HIF-1α) is a key sensor of tissue hypoxia and a central regulator of angiogenesis. Under normoxic conditions, HIF-1α is rapidly degraded via the proteasomal pathway following prolyl hydroxylation. However, under reduced oxygen availability, hydroxylation is inhibited, leading to stabilization of the protein, its translocation to the nucleus, and formation of a complex with HIF-1β. This complex binds to hypoxia-responsive elements (HREs) in gene promoters and activates the transcription of numerous angiogenic factors, including VEGF-A, FGF-2, ANGPT, and SDF-1/CXCL12, thereby promoting endothelial cell proliferation and migration, tubular structure formation, and recruitment of EPCs to ischemic sites [91,92]. Experimental models of ischemia have demonstrated that in vivo stabilization of HIF-1α enhances the formation of a functional capillary network and facilitates vascular remodeling, confirming its critical role in both physiological and therapeutic angiogenesis [93]. Thus, HIF-1α acts as a central regulator of vascular adaptation to hypoxia, integrating signals from multiple angiogenic factors and ensuring coordinated restoration of blood flow in ischemic tissues [93].

In patients with CLTI, HIF-1α levels are significantly elevated in ischemic skeletal muscle, accompanied by increased expression of its target genes, including VEGF, SDF-1/CXCL12, FGF-2, and ANGPT. Activation of HIF-1α mediates both pro-angiogenic effects and mobilization of endothelial progenitor cells from the bone marrow, thereby contributing to new capillary formation and stabilization of the vascular network. Nevertheless, in chronic and severe ischemia, endogenous activation of HIF-1α is often insufficient to restore adequate limb perfusion, which provides a rationale for therapeutic strategies aimed at enhancing HIF-1α-dependent angiogenic responses, including gene therapy [94,95].

Particular attention should be paid to how metabolic stress, especially in patients with diabetes mellitus, affects hypoxia signaling pathways. Chronic metabolic stress, particularly in diabetes mellitus, may profoundly dysregulate HIF-1α–dependent hypoxia signaling despite the presence of tissue ischemia and hypoxia. Hyperglycemia, oxidative stress, mitochondrial dysfunction, and accumulation of advanced glycation end products have been shown to impair HIF-1α stabilization and transcriptional activity, thereby reducing the expression of downstream angiogenic mediators such as VEGF. As a consequence, diabetic tissues often exhibit defective adaptive angiogenesis, impaired collateral vessel formation, and delayed tissue repair. Experimental studies further suggest that high-glucose conditions may enhance proteasomal degradation of HIF-1α and disrupt normal hypoxia-responsive signaling pathways, contributing to endothelial dysfunction and chronic ischemic injury. This impaired HIF-1α response is considered one of the key molecular mechanisms underlying poor wound healing and insufficient neovascularization in diabetic vascular disease [96].

**Figure 2:**
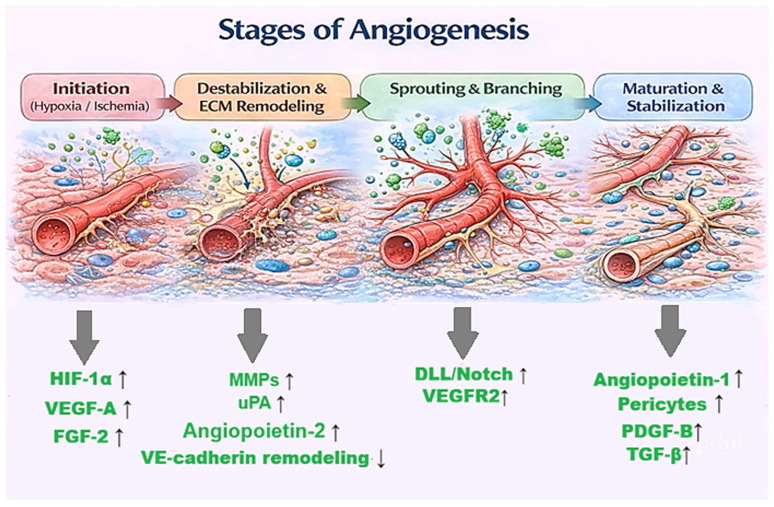
The most important angiogenic factors and their role in angiogenesis. Abbreviations: HIF-1α—hypoxia-inducible factor 1-alpha; VEGF-A—vascular endothelial growth factor A; FGF-2—fibroblast growth factor 2; MMPs—matrix metalloproteinases; uPA—urokinase-type plasminogen activator; VE-cadherin—vascular endothelial cadherin; DLL/Notch—Delta-like ligand/Notch signaling pathway; VEGFR2—vascular endothelial growth factor receptor 2; PDGF-B—platelet-derived growth factor B; TGF-β—transforming growth factor beta. The “Stages of Angiogenesis” illustration presented in Figure 2 is an original figure created by the authors specifically for this manuscript and was not adapted from any previously published source.

In summary, angiogenesis is a complex, multistep process regulated by a network of interconnected molecular signals that coordinate the activation, migration, proliferation, and differentiation of vascular wall cells (Figure 2). HIF-1α serves as the central regulator of the hypoxic response, inducing the expression of key angiogenic mediators. Among these, VEGF plays a leading role in promoting vascular growth and permeability, while other factors, including FGF, HGF, and SDF-1, regulate cell migration, extracellular matrix remodeling, and progenitor cell recruitment. At later stages of vascular network formation, stabilizing and remodeling signals such as ANGPT and ANG become critically important. The coordinated action of these factors establishes a dynamic regulatory system that enables vascular adaptation to both physiological and pathological conditions. At the same time, the efficiency of the angiogenic response in chronic ischemia is limited by pronounced endothelial dysfunction, oxidative stress, and metabolic disturbances. This underscores the need for further investigation of the molecular mechanisms governing angiogenesis and for the development of combined therapeutic strategies aimed at enhancing reparative processes and improving clinical outcomes in this patient population.

## 7. Gene Therapy

Gene therapy offers a promising therapeutic strategy for patients with CLTI and represents a promising approach to the treatment of lower limb ischemia, with the potential to induce therapeutic neoangiogenesis. To date, various methods for delivering gene-based therapeutics to ischemic limbs have been developed, including both viral and non-viral approaches [97,98,99].

In the context of the limited efficacy of the endogenous angiogenic response in CLTI, gene therapy is of particular interest as a strategy aimed at targeted enhancement of the expression of protective and angiogenic factors within ischemic tissues. This approach enables localized and sustained production and vector-mediated delivery of therapeutic molecules, including VEGF, FGF, HGF, and others, thereby overcoming limitations associated with their short half-life and systemic side effects observed with exogenous protein administration. This section reviews current gene delivery strategies for lower limb ischemia, their mechanisms of action, advantages, limitations, and outcomes of clinical application.

**Non-viral delivery methods**

**Plasmids**

Plasmid-based gene delivery represents one of the safest and most technically straightforward approaches in gene therapy for lower limb ischemia. Plasmid DNA does not integrate into the host cell genome, thereby markedly reducing the risk of insertional mutagenesis and carcinogenesis, while also ensuring low immunogenicity and enabling repeated administration—an important consideration in the treatment of chronic non-oncological diseases. In addition, plasmid vectors are relatively simple to manufacture under GMP conditions and allow flexible design of mono- and multigene expression cassettes, including combinations of angiogenic factors as well as bi- or polycistronic constructs [100]. Clinical studies have generally demonstrated favorable tolerability and an acceptable safety profile of plasmid-based therapies. However, their therapeutic efficacy has remained variable, with several large randomized trials reporting only modest or inconsistent clinical benefit. These limitations are likely related, at least in part, to the relatively low efficiency of in vivo transfection and the transient nature of transgene expression following administration. Consequently, plasmid-based approaches often require enhanced delivery strategies, such as electroporation or micro-/nanotechnology-based systems, as well as repeated administrations of gene constructs to achieve sustained therapeutic effects. Therefore, interpretations of their clinical potential should remain cautious and balanced.

In preclinical studies, administration of combinations of plasmids encoding *VEGF, ANG, GDNF, FGF*-2, or *HGF* into ischemic skeletal muscle resulted in a significant increase in the expression of the respective factors, improved blood perfusion, and stimulation of capillary formation, with combined delivery of *VEGF*- and *HGF*-encoding plasmids producing more pronounced effects compared to single-plasmid administration [101,102]. Intradermal administration of an *FGF*2-encoding plasmid followed by skin electroporation in rats led to a marked increase in *FGF*2 expression and a twofold rise in capillary density in the ischemic limb, which was associated with improved blood flow [103].

In experiments involving intramuscular delivery of an *HGF*-encoding plasmid in rats and rabbits, a substantial enhancement of angiogenesis and revascularization in ischemic limb muscles was observed compared to controls [104,105]. Results from intramuscular administration of a *VEGF*165-encoding plasmid in a rabbit hindlimb ischemia model demonstrated higher capillary and arteriolar density following double administration compared to a single injection, underscoring the importance of sustained transgene expression [106]. Finally, intramuscular delivery of a combined bicistronic plasmid encoding *VEGF*165 and *HGF* in a mouse hindlimb ischemia model showed that co-delivery of genes can influence tissue and mitochondrial regeneration, although these effects depend on concomitant metabolic disturbances in the animals [107].

According to recent reviews, further investigation of growth factors such as VEGF, HGF, and FGF requires optimization of delivery strategies, as well as the establishment of a supportive tissue microenvironment capable of sustaining stable angiogenesis and arteriogenesis [108]. More comprehensive approaches involving the sequential use of plasmids encoding *VEGF, HGF*, and *ANG* have demonstrated improved perfusion and clinical parameters in experimental models, including those with diabetic pathology, underscoring the need for multifactorial strategies in preclinical research [109].

Clinical studies indicate that plasmid-based therapy can improve perfusion parameters and promote ulcer healing in patients with critical limb ischemia, while maintaining a favorable safety profile. However, the magnitude of therapeutic efficacy varies, ranging from moderate improvements to a lack of statistically significant differences in large randomized trials [110]. For example, early-phase clinical studies in patients with critical limb ischemia showed that local intramuscular administration of a *VEGF*165-encoding plasmid was well tolerated and not associated with serious adverse events; however, it failed to produce significant improvements in key clinical outcomes, such as reduction in amputation rates or restoration of perfusion [48,111].

Kibbe et al. (2016) demonstrated in clinical trials that complete ulcer healing, a significant (>50%) reduction in ulcer area, and a marked increase in tissue oxygenation (TcPO_2_) were observed following administration of an *HGF*-encoding plasmid compared to a placebo [112]. Ajroud-Driss et al. investigated the angiogenic effects of plasmid DNA (VM202), expressing the *HGF*723 and *HGF*728 isoforms, in patients with diabetic peripheral neuropathy complicated by limb ischemia. In phase I clinical trials, VM202 treatment was safe, well tolerated, and showed potential therapeutic benefits in this patient population [113]. Similarly, phase II studies confirmed the safety and tolerability of this therapy. Intramuscular administration of an *HGF*-encoding plasmid resulted in a statistically significant improvement in ulcer healing in patients with critical ischemia, while maintaining a favorable safety and tolerability profile [112].

Positive effects have also been reported following intramuscular administration of a bicistronic plasmid construct encoding *VEGF* and *HGF* in patients with limb ischemia complicated by diabetes mellitus. Standard therapy for CLTI includes antiplatelet agents, statins, and surgical revascularization; however, many patients have significant limitations for surgical intervention, highlighting the need for alternative treatment approaches. It has been shown that 90 days after intramuscular administration of the *VEGF/HGF* plasmid, patients exhibited increased serum VEGF levels and ankle-brachial index (ABI), along with a significant reduction in rest pain. CT angiography revealed characteristic progressive changes associated with diabetic microangiopathy, whereas angiography demonstrated the formation of new collateral vessels and improved distal blood flow. Importantly, gene therapy did not adversely affect the course of diabetes, as fasting glucose levels remained within the range of 6.5–8 mmol/L [26].

Hammad et al. conducted a multicenter, randomized, double-blind, placebo-controlled phase IIb trial involving 109 patients following successful revascularization. Patients received intramuscular injections of an *SDF*1-encoding plasmid twice at a 3-month interval. After 6 months, wound healing rates were approximately 31–33% across all groups, and the ABI increased significantly; however, major adverse limb events (MALE) were not reduced. Thus, the addition of plasmid-based therapy to revascularization did not improve clinical outcomes at 6 months [114]. Large-scale clinical trials of plasmid-based gene therapies, including TALISMAN and TAMARIS, have been conducted (ClinicalTrials.gov, NCT00368797; NCT00566657) [115,116,117,118,119]. The results of the TAMARIS trial were highly disappointing, as the study failed to demonstrate any clinical benefit of gene therapy in patients with critical limb ischemia. In contrast to earlier, smaller studies such as TALISMAN, TAMARIS showed no significant improvement in amputation-free survival or other major clinical endpoints [116].

In 2011, a plasmid-based therapeutic agent, Neovasculgen^®^ (Moscow, Russia), containing the *VEGF*165 gene, was approved in Russia. The greatest clinical benefit was observed in patients with stage III disease, where pain-free walking distance increased by 683%. No adverse events associated with angiogenic therapy were reported [120]. Five-year follow-up results of Neovasculgen therapy have since been published. The treatment was well tolerated, with no increase in the incidence of major cardiovascular events, malignancies, or visual disturbances. Pain-free walking distance increased from approximately 106 to 384 m, limb salvage reached about 95% (compared to ~67% in the control group), and the ABI remained elevated. These findings indicate that the therapeutic effect of angiogenesis stimulation is sustained for at least five years following a course of Neovasculgen therapy [121]. Although long-term follow-up studies of Neovasculgen have reported encouraging clinical outcomes, these findings should be interpreted cautiously due to the limited availability of large-scale, independently validated international randomized studies confirming its efficacy and long-term benefit.

Plasmid-mediated gene delivery represents a safe and accessible approach to gene therapy for lower limb ischemia, offering several advantages as outlined above. These properties make plasmid constructs particularly attractive for the treatment of chronic vascular diseases, where prolonged and controlled stimulation of angiogenesis is required. Preclinical studies consistently demonstrate that plasmid vectors encoding angiogenic and neurotrophic genes (*VEGF*, *HGF*, *FGF*, *ANG*, *GDNF*) can enhance neovascularization, improve tissue perfusion, and stimulate regenerative processes. Notably, the highest efficacy is achieved with the combined delivery of multiple genes, supporting the concept of a multifactorial and simultaneous intervention in the ischemic pathological process.

Despite promising results in experimental models, clinical studies have demonstrated more modest and variable effects. Plasmid-based gene therapy is generally characterized by a strong safety profile and good tolerability; however, its impact on key clinical outcomes, such as reductions in amputation rates or mortality, remains limited. Certain agents and approaches, including those based on *HGF* or *VEGF* genes, as well as the approved drug Neovasculgen, have shown improvements in functional and perfusion-related parameters. Nevertheless, large randomized trials (e.g., TAMARIS) have not confirmed significant clinical benefit. Accordingly, large meta-analyses of randomized gene therapy trials have not demonstrated statistically significant changes in ABI, pain, or amputation rates; however, treatment was associated with improved ulcer healing and increased tissue oxygenation, indicating potential therapeutic efficacy and the need for further investigation [119]. Several mechanistic and translational factors may explain this discrepancy. First, TAMARIS enrolled patients with advanced CLTI, in whom endothelial dysfunction, microvascular rarefaction, inflammation, oxidative stress, and impaired nitric oxide bioavailability are often already severe. In such a setting, the ischemic tissue may no longer be capable of responding adequately to a single angiogenic stimulus. This is particularly relevant in patients with diabetes, where endothelial cells, pericytes, smooth muscle cells, and resident progenitor populations exhibit impaired responsiveness to VEGF, FGF, HGF, and other pro-angiogenic cues. Thus, even sufficient local expression of a therapeutic transgene may not translate into effective vascular remodeling if downstream signaling pathways are dysfunctional. Second, the biology of chronic human ischemia differs substantially from acute experimental ischemia models. Preclinical models typically involve relatively young animals, abrupt arterial ligation, preserved regenerative capacity, and limited comorbidity. By contrast, patients with CLTI often present with long-standing atherosclerosis, diabetes, infection, tissue necrosis, renal dysfunction, neuropathy, and impaired wound healing. These factors create a hostile tissue microenvironment in which angiogenesis, arteriogenesis, extracellular matrix remodeling, and inflammatory resolution are simultaneously compromised. Third, vector-related factors may have limited efficacy. Plasmid-based gene transfer is generally safe and well tolerated, but transfection efficiency in ischemic skeletal muscle is relatively low and expression is usually transient. Therefore, the dose, injection pattern, tissue distribution, and duration of transgene expression may have been insufficient to generate sustained and spatially coordinated vascular growth. Therapeutic angiogenesis likely requires not only increased expression of one growth factor, but also appropriate timing, concentration gradients, and interaction with stabilizing pathways such as PDGF-mediated mural cell recruitment and extracellular matrix remodeling. Fourth, patient heterogeneity and disease stage are critical. CLTI is not a uniform disease entity: patients differ in the extent of macrovascular occlusion, collateral capacity, microvascular damage, diabetes status, renal function, infection burden, ulcer severity, and background medical therapy. Such heterogeneity may dilute treatment effects in large trials, particularly if only a biologically responsive subgroup benefits. The negative TAMARIS result therefore does not necessarily exclude biological activity of gene therapy, but suggests that broad, unselected application of single-factor angiogenic therapy is unlikely to produce robust clinical benefit in advanced CLTI.

These limitations have stimulated the development of next-generation therapeutic strategies aimed at overcoming the insufficient efficacy of single-factor angiogenic therapy. An important direction for improving the efficacy of plasmid-based gene therapy lies in optimizing gene delivery methods, as well as in the development of bicistronic constructs that provide synergistic effects of angiogenic and neurotrophic factors. Combination strategies involving the delivery of multiple genes have demonstrated more pronounced effects in both preclinical and clinical studies, including enhanced angiogenesis, improved tissue perfusion, and better patient quality of life.

***Liposomes***

Liposomes are spherical vesicles composed of one or more phospholipid bilayers. Owing to their biocompatibility, their ability to encapsulate both hydrophilic and hydrophobic molecules, and their capacity to protect nucleic acids from degradation, liposomes are widely used as delivery systems for drugs and genetic material. These properties make them highly promising nonviral vectors, including for applications aimed at stimulating angiogenesis in CLTI and for the local delivery of therapeutic genes.

In a rabbit model of acute hindlimb ischemia, transfection of skeletal myoblasts with a *VEGF*165-encoding plasmid using cholesterol + DOTAP liposomes (CD liposomes) was shown to effectively stimulate neovascularization. Optimized transfection resulted in *VEGF*165 expression lasting up to 18 days, with a peak on day 2. Following transplantation of CD-p*VEGF*165, a significant increase in collateral vessel formation, capillary density (CD31), and blood flow was observed compared to control groups [122]. In another study using a similar rabbit model, delivery of a *VEGF*165-encoding plasmid via cationic lipids led to a marked increase in capillary density, enhanced collateral vessel formation, and improved perfusion of ischemic tissue [123]. These findings support the efficacy of liposome-mediated *VEGF* gene delivery for the induction of therapeutic angiogenesis in limb ischemia.

Lipid–polymer nanoparticles (FLNPs) represent a so-called membrane-fusogenic and nucleus-targeted “all-in-one” platform for gene delivery in ischemic conditions, including limb ischemia. Encapsulation of an *HGF*-encoding plasmid together with a catalase-encoding plasmid (p*CAT*) within *HGF/CAT* FLNPs enables the simultaneous enhancement of angiogenesis and detoxification of hydrogen peroxide, thereby increasing cellular resistance to oxidative stress in vitro and promoting restoration of perfusion, limb function, and reduction in gangrene risk in a mouse hindlimb ischemia model [124].

It has also been demonstrated that delivery of the *FGF*2 gene using proteoliposomes carrying the co-receptor syndecan-4 significantly enhances angiogenic and arteriogenic responses in a limb ischemia model. Liposomally incorporated syndecan-4 improved cellular uptake of *FGF*2, enhanced endothelial cell proliferation, migration, and vascular network formation in vitro, and resulted in more pronounced neovascularization and perfusion recovery in ischemic rat hindlimbs compared to *FGF*2 monotherapy. These findings indicate that co-delivery of a co-receptor alongside a growth factor may increase therapeutic efficacy in limb ischemia [125].

Overall, liposomal delivery systems represent a promising and safe direction in nonviral gene therapy for lower limb ischemia due to their biocompatibility, low immunogenicity, and lack of genomic integration. They provide protection and efficient localized delivery of genes, serving as an alternative to viral vectors. Preclinical data demonstrate that liposome-mediated delivery of therapeutic genes stimulates angiogenesis, increases capillary density, and improves tissue perfusion. Enhanced efficacy can be achieved using advanced systems such as lipid–polymer nanoparticles and proteoliposomes, which enable simultaneous modulation of angiogenesis, cell survival, and regeneration. However, despite encouraging results from a limited number of preclinical studies employing liposomal delivery of angiogenic genes, translation into routine clinical practice in humans has not yet been achieved.

***Viral delivery approaches***

The most commonly used viral vectors for therapeutic angiogenesis include adeno-associated viruses (AAVs), adenoviruses (Ads), and lentiviruses (LVs). AAV vectors are characterized by relatively low immunogenicity and stable episomal expression, whereas Ad vectors provide high transduction efficiency but induce more pronounced immune responses. LV vectors integrate into the host genome, enabling long-term transgene expression but carrying a theoretical risk of insertional mutagenesis. Advances in viral vector engineering and gene expression control have enabled the development of safe and effective clinical-grade viral gene therapy products [126]. In contrast to plasmids, viral vectors demonstrate high in vivo transduction efficiency and can provide sustained transgene expression in target cells, making them key tools for angiogenic gene therapy in lower limb ischemia, although careful optimization of clinical delivery protocols is required. Although viral vectors provide substantially higher transduction efficiency compared with non-viral approaches, their clinical application remains complicated by host immune responses, inflammatory toxicity, and biosafety concerns [127].

One of the major limitations involves pre-existing immunity against viral capsids. Neutralizing antibodies against Ad and AAV vectors are highly prevalent in the general population and may significantly reduce vector transduction efficiency and duration of transgene expression [128]. In addition, first-generation Ad vectors may trigger strong innate immune activation and inflammatory cytokine release, including TNF-α, IL-6, and IFN-γ, thereby contributing to endothelial dysfunction and accelerated vector clearance [129]. These effects are particularly relevant in CLTI patients, who frequently exhibit chronic systemic inflammation and severe vascular pathology.

Another important challenge concerns the safety of angiogenic transgene expression itself. Excessive VEGF activity may increase vascular permeability and promote tissue edema. In the RAVE trial, dose-dependent peripheral edema was observed following intramuscular *VEGF* gene delivery, likely reflecting VEGF-mediated disruption of endothelial barrier integrity and increased plasma leakage within ischemic tissues [130]. Additional concerns involve vector persistence, shedding, and genotoxicity. LV vectors provide stable long-term expression but carry a theoretical risk of insertional mutagenesis due to genomic integration [131]. Consequently, regulatory agencies such as the FDA and EMA require extensive evaluation of biodistribution, vector shedding, long-term safety, and immunogenicity during clinical development [132]. Together, these limitations highlight the need for safer and more controllable next-generation gene delivery systems with improved tissue specificity, reduced immunogenicity, and regulated transgene expression.

AAV vectors exhibit strong tropism for endothelial and muscle cells and enable sustained expression of angiogenic factors such as VEGF. In preclinical models of CLTI, AAV2-mediated *VEGF* delivery—including the study by Shimpo et al. (2002)—stimulated angiogenesis, increased capillary density, and improved regional perfusion in ischemic skeletal muscle in rats, with moderate immune responses and minimal systemic adverse effects [133,134]. In another study, an AAV vector with hypoxia-inducible *VEGF* expression effectively stimulated angiogenesis in a mouse hindlimb ischemia model. This system restricts *VEGF* expression predominantly to ischemic tissues, resulting in improved perfusion and vascular regeneration while reducing the risk of adverse effects associated with uncontrolled angiogenic stimulation [117]. Co-delivery studies using AAV2-VEGF and AAV2-FGF have also been conducted. This combined approach stimulated both angiogenesis and arteriogenesis, increasing capillary density and collateral vessel formation, which led to improved blood flow and accelerated recovery of ischemic necrotic tissue compared to VEGF monotherapy [135].

Ad vectors provide rapid and robust transgene expression, which is particularly advantageous for short-term stimulation of angiogenesis. However, their higher immunogenicity limits repeated administration and may reduce therapeutic efficacy; they are also associated with adverse effects, such as limb edema (likely due to overexpression of angiogenic factors) [136]. Administration of Ad-VEGF has been shown to induce sustained increases in capillary diameter and enhanced angiogenesis in ischemic muscle. However, despite pronounced morphological changes in the vascular network, these effects were not accompanied by improved muscle regeneration or functional recovery in hyperlipidemic mice, and significant limb edema was observed [137]. In another study using a rabbit hindlimb ischemia model, Ad-VEGF delivery to ischemic skeletal muscle resulted in marked early-stage angiogenesis, with increased capillary density and improved perfusion at the injection site compared to controls. However, the effect was transient, correlating with short-lived transgene expression, and was accompanied by increased vascular permeability, mild tissue edema, and evidence of ectopic transgene expression [138]. These findings suggest that although adenoviral vectors can stimulate angiogenesis in limb ischemia models, the functional stability and durability of the neovascular network remain limited, which must be considered in therapeutic design.

At the same time, Sarkar et al. demonstrated that intramuscular administration of Ad-HIF-1α significantly enhanced both angiogenesis and arteriogenesis, improved perfusion, and reduced ischemic tissue damage in a mouse model of acute limb ischemia with diabetes [139]. The regenerative potential of direct gene therapy using recombinant adenoviruses encoding *VEGF, GDNF*, and *ANG* was evaluated in a rat model of chronic hindlimb ischemia. A single combined injection of Ad5-VEGF, Ad5-ANG, and Ad5-GDNF stimulated muscle regeneration, increasing the number of centrally nucleated muscle fibers and blood vessels, indicating a positive synergistic effect [140].

LVs are characterized by their ability to integrate into the host genome, providing long-term expression and efficient transduction of non-dividing cells. This makes them promising for sustained angiogenic stimulation in ischemia models, although integration-related risks necessitate strict safety control [141]. In mouse hindlimb ischemia models, intramuscular injection of a lentivirus encoding the transcription factor Ets variant 2 (ETV2) stimulated endothelial cell proliferation, enhanced angiogenesis, and improved perfusion of ischemic tissue, accompanied by reduced necrosis and increased limb survival [142]. The same authors demonstrated that combined LV-ETV2 and HGF therapy significantly enhanced angiogenesis, promoted tissue and perfusion recovery, and reduced limb damage compared to monotherapy [143]. Wang et al. showed that lentiviral delivery of the *EPAS*1 gene (endothelial PAS domain protein 1) into ischemic hindlimb muscle in rats resulted in significant improvements in blood circulation and limb function, including histological recovery, improved gait, and increased blood flow as measured by laser Doppler analysis [144].

Turning to the limited number of clinical studies, two early-phase trials have demonstrated safety and tolerability but have not shown clear efficacy. The RAVE trial—a phase II double-blind, placebo-controlled study—was designed to evaluate the efficacy and safety of intramuscular Ad-VEGF121 injection in patients with CLTI. The results showed that a single administration of Ad-VEGF121 did not improve pain-free walking time, ABI, or quality of life [130]. In another large randomized double-blind placebo-controlled trial (ClinicalTrials.gov, NCT00117650), the efficacy of Ad-HIF-1α was assessed in patients with CLTI. No significant differences were observed between treatment and placebo groups in pain-free walking distance, ABI, or quality-of-life measures. Thus, Ad-HIF-1α gene therapy did not demonstrate statistically significant improvement in clinical outcomes [145].

Despite the progress achieved to date, safe and routine clinical application of viral gene delivery remains a distant prospect. One of the key risks of gene therapy is the induction of neovascularization in non-target tissues. Additional limitations include the potential for inflammatory reactions and the theoretical risk of insertional mutagenesis and oncogenic transformation. VEGF is a potent mediator of vascular permeability and may promote the formation of immature and structurally unstable neovessels lacking adequate mural cell coverage, thereby increasing the risk of tissue edema and vascular leakage [146]. In addition, VEGF signaling has been linked to endothelial barrier disruption through destabilization of VE-cadherin-dependent intercellular junctions, which may further enhance vascular permeability and inflammatory cell infiltration [147]. Excessive or uncontrolled *VEGF* overexpression may also contribute to aberrant angiogenesis characterized by disorganized vascular architecture, impaired vessel maturation, and reduced long-term functional stability of newly formed capillaries. Furthermore, VEGF-mediated endothelial activation may interact with thrombo-inflammatory pathways by promoting leukocyte adhesion, platelet activation, and local inflammatory responses, potentially exacerbating microvascular dysfunction in chronically ischemic tissues. These mechanisms may partially explain the occurrence of peripheral edema and other vascular adverse events observed in certain clinical studies of VEGF-based therapeutic angiogenesis. These considerations are particularly relevant in patients with CLTI, who already exhibit a high baseline risk of thrombosis, endothelial dysfunction, and systemic vascular inflammation. Experimental and histopathological studies suggest that VEGF-driven intraplaque neovascularization may contribute to plaque vulnerability, as newly formed microvessels are frequently fragile, disorganized, and prone to leakage or intraplaque hemorrhage [148]. Therefore, future therapeutic strategies should focus not only on stimulating angiogenesis, but also on promoting vascular maturation and long-term vessel stability in order to minimize potential pro-inflammatory and pro-thrombotic complications.

Preclinical and clinical studies of angiogenic gene therapy using viral vectors have yielded mixed results, combining both positive and negative outcomes, thereby limiting widespread clinical implementation and restricting these approaches primarily to no-option patients. It is important to note that the number of studies employing viral vectors is substantially lower than those using plasmid-based constructs, with many still in early stages (preclinical or phase I). Moreover, two large clinical trials involving viral vectors failed to demonstrate clinical efficacy, whereas several preclinical studies using similar systems have reported encouraging results.

One of the central unresolved issues in the field of therapeutic angiogenesis for CLTI is the persistent discrepancy between promising preclinical outcomes and the limited efficacy observed in human clinical trials. Numerous experimental studies demonstrated enhanced neovascularization, improved perfusion, and tissue preservation following delivery of angiogenic genes. However, these encouraging findings have not consistently translated into meaningful clinical benefit in randomized trials.

One of the major reasons underlying translational failure is the fundamental difference between experimental ischemia models and the pathophysiology of human CLTI. Most preclinical studies utilize acute hindlimb ischemia models in young, otherwise healthy rodents, in which femoral artery ligation or excision induces a rapid and severe reduction in blood flow [149,150]. In these models, endogenous regenerative capacity remains largely preserved, and collateral vessel formation occurs rapidly in response to ischemic stimuli. In contrast, human CLTI represents a chronic, progressive, multifactorial atherosclerotic disease that develops over decades and is strongly associated with aging, diabetes mellitus, smoking, hyperlipidemia, chronic inflammation, renal dysfunction, and endothelial impairment [3,8]. Patients with CLTI frequently exhibit diffuse arterial calcification, microvascular dysfunction, neuropathy, oxidative stress, chronic inflammation, and impaired tissue regeneration, all of which substantially reduce angiogenic responsiveness [9]. Consequently, the biological environment in human ischemic tissues differs profoundly from that observed in experimental models. Furthermore, rodent models rarely reproduce the systemic inflammatory burden, endothelial senescence, metabolic dysregulation, and severe microvascular rarefaction characteristic of advanced human vascular disease. Aging itself significantly impairs endothelial progenitor cell mobilization, nitric oxide signaling, mitochondrial function, and growth-factor responsiveness [151]. Diabetes-associated advanced glycation end products, endothelial dysfunction, and persistent inflammation additionally suppress angiogenic signaling pathways and impair vascular repair mechanisms, thereby limiting the therapeutic activity of VEGF- and FGF-based interventions [152]. Impaired macrophage polarization, extracellular matrix remodeling, and reduced regenerative potential of chronically ischemic skeletal muscle may further compromise stable neovascularization and tissue recovery.

Another important limitation is that preclinical models generally involve isolated arterial occlusion without the extensive collateral vascular remodeling, tissue fibrosis, and long-standing ischemic damage commonly observed in patients with CLTI. In addition, clinical populations are highly heterogeneous with respect to disease severity, duration of ischemia, smoking status, metabolic comorbidities, concomitant pharmacotherapy, and baseline collateral vessel development, all of which may substantially influence therapeutic responsiveness and contribute to variable clinical outcomes. Vector-related limitations, including transient transgene expression, insufficient tissue penetration, low transfection efficiency, and pre-existing immunity against viral vectors, may further reduce therapeutic efficacy in clinical settings. As a result, therapeutic angiogenesis appears considerably more effective in animal studies than in clinical reality.

The majority of early clinical trials were based on delivery of a single angiogenic factor, most commonly VEGF, FGF, HGF, HIF-1α, or SDF-1. Although these molecules play important roles in vascular development and endothelial activation, angiogenesis is an exceptionally complex and tightly regulated biological process involving coordinated interactions among endothelial cells, smooth muscle cells, pericytes, extracellular matrix components, inflammatory mediators, and mechanical stimuli [10]. VEGF-based therapies demonstrated substantial promise in experimental studies due to their ability to stimulate endothelial proliferation and increase vascular permeability. However, excessive or poorly regulated VEGF expression may also result in the formation of immature, unstable, and hyperpermeable vessels associated with tissue edema and limited long-term perfusion improvement [153]. Similarly, FGF-mediated angiogenesis may enhance capillary density without necessarily producing durable and functional vascular networks. These observations suggest that stimulation of endothelial proliferation alone may be insufficient to achieve stable and functional vascular regeneration in chronic ischemic tissues. Clinical experience suggests that isolated stimulation of angiogenesis alone is insufficient to restore tissue integrity in advanced ischemic disease. Effective tissue regeneration likely requires simultaneous modulation of arteriogenesis, inflammation, extracellular matrix remodeling, neurovascular repair, and skeletal muscle regeneration [18]. This limitation partially explains the growing interest in combinatorial and multimodal regenerative approaches.

Another major challenge involves the limited efficiency and duration of gene delivery systems used in clinical studies. Non-viral vectors, including naked plasmid DNA and liposomal formulations, are generally considered safe and well tolerated but demonstrate low transfection efficiency and transient gene expression in ischemic tissues [154]. Rapid degradation of plasmid DNA, insufficient cellular uptake, and poor tissue retention substantially reduce therapeutic efficacy. Viral vectors, including Ad and AAV systems, provide higher transduction efficiency and stronger transgene expression. Nevertheless, their clinical use is restricted by immunogenicity, inflammatory responses, dose limitations, and concerns regarding long-term safety [155]. Ad vectors may induce significant innate immune activation and transient systemic inflammatory reactions, while pre-existing neutralizing antibodies against AAV vectors may limit transduction efficiency in humans [156]. In addition, delivery approaches themselves remain suboptimal. Intramuscular injections may produce heterogeneous transgene distribution and limited penetration into ischemic tissues, whereas intra-arterial administration can be compromised by impaired distal perfusion and severe vascular obstruction. Tissue-specific and controllable gene expression systems remain insufficiently developed for clinical application.

Clinical trials investigating therapeutic angiogenesis have also been complicated by substantial heterogeneity among patient populations. Many studies included patients with advanced CLTI who had severe comorbidities, extensive tissue damage, and limited endogenous regenerative capacity [115]. Such patients may be biologically incapable of mounting sufficient angiogenic responses even when exposed to exogenous growth factors. In addition, differences in disease stage, lesion distribution, diabetes prevalence, smoking status, concomitant pharmacotherapy, and prior revascularization procedures significantly complicate interpretation of trial outcomes and inter-study comparisons. Small sample sizes and inconsistent inclusion criteria further limit statistical power. The randomized RAVE and TAMARIS trials illustrate these challenges. Despite evidence of biological activity and acceptable safety profiles, neither study demonstrated significant improvements in major clinical endpoints such as amputation-free survival or reduction in major adverse limb events. These findings underscore the complexity of translating angiogenic stimulation into clinically meaningful benefit.

Another important issue concerns the selection of clinical endpoints in therapeutic angiogenesis trials. Many early studies relied on surrogate parameters such as ABI, TcPO2, angiographic vessel density, or pain scores. However, improvements in these surrogate markers do not necessarily correlate with durable limb salvage or functional recovery [157]. Moreover, angiogenesis is a relatively slow biological process, whereas patients with advanced CLTI frequently require urgent restoration of tissue perfusion. In many cases, irreversible tissue necrosis and chronic inflammation may already be too advanced for angiogenic therapies alone to reverse disease progression. The complexity of human ischemic disease suggests that future clinical studies should incorporate multidimensional endpoints, including tissue regeneration, wound healing, functional recovery, quality of life, microvascular perfusion, and long-term limb preservation.

Collectively, these findings suggest that the limited success of therapeutic angiogenesis in clinical settings does not reflect failure of the concept itself, but rather the biological complexity of chronic ischemic disease and the inadequacy of early-generation therapeutic strategies. The limitations of first-generation angiogenic gene therapy have stimulated the development of more sophisticated regenerative approaches. Future strategies will likely require combined modulation of angiogenesis, arteriogenesis, inflammation, and tissue repair rather than isolated delivery of single growth factors. Several emerging approaches are currently under investigation, including multicistronic vectors encoding multiple synergistic growth factors, tissue-specific promoters, inducible gene expression systems, exosome-mediated delivery, biomaterial-assisted gene transfer, and combined gene–cell therapy platforms involving mesenchymal stromal cells or endothelial progenitor cells [158]. Biomaterial scaffolds and hydrogel-based delivery systems may improve local retention and controlled release of angiogenic factors while simultaneously supporting tissue regeneration. In addition, advances in genome engineering and RNA-based therapeutics may enable more precise regulation of therapeutic gene expression and reduction in off-target effects. However, successful clinical translation will require improved animal models that better reproduce chronic human vascular disease, standardized clinical endpoints, optimized patient stratification, and long-term safety evaluation. Overall, the experience accumulated over the past two decades demonstrates that therapeutic angiogenesis remains biologically promising but clinically challenging. The failure of early-generation strategies does not necessarily invalidate the concept itself; rather, it highlights the need for more comprehensive regenerative approaches capable of addressing the multifactorial nature of chronic ischemic disease. Major clinical trials of gene therapy for lower limb ischemia are summarized in Table 1.

## 8. Conclusions

Clinical investigations of gene therapy for chronic and critical lower limb ischemia have demonstrated that both viral and non-viral vectors are capable of mediating biologically relevant expression of angiogenic factors in vivo; however, in most cases this has not translated into durable clinical benefit. The negative or neutral outcomes of large-scale randomized controlled trials, most notably RAVE and TAMARIS, underscore the intrinsic limitations of first-generation angiogenic monotherapy strategies based on uncontrolled or transient expression of individual growth factors.

The cumulative clinical evidence indicates that therapeutic angiogenesis in limb ischemia represents a multifactorial process extending far beyond the simple induction of neovascularization. Insufficient arteriogenesis, pathological vascular permeability, persistent inflammatory activation, and neurodegenerative alterations within ischemic tissues collectively limit the clinical efficacy of single-component therapeutic approaches, irrespective of the delivery vector employed.

In this context, future progress in gene therapy will likely depend on the development of next-generation strategies incorporating optimized viral and non-viral vectors capable of controlled, spatially and temporally regulated transgene expression, together with combinatorial targeting of angiogenesis, arteriogenesis, and tissue regeneration. Recent advances in therapeutic angiogenesis have focused on next-generation gene delivery platforms designed to improve tissue specificity, biosafety, and temporal control of transgene expression. Among these, mRNA-based angiogenic therapy using lipid nanoparticle (LNP) formulations has emerged as a promising non-viral strategy, enabling transient yet efficient expression of pro-angiogenic factors without the risk of genomic integration. Recent studies in murine hindlimb ischemia and critical limb ischemia models demonstrated that *VEGF-*, *ETV2*-, or *SDF-1α*-encoding mRNA delivered via LNPs significantly improved perfusion recovery, angiogenesis, and vascular regeneration [162,163,164].

Extracellular vesicles and exosomes are also attracting considerable attention as naturally derived carriers capable of delivering nucleic acids, proteins, and regulatory RNAs with low immunogenicity and improved biocompatibility [165]. In parallel, CRISPR/dCas9-based epigenetic activation approaches offer the possibility of inducing endogenous angiogenic pathways through targeted transcriptional activation of loci such as VEGF-A and HIF-1α, thereby providing more physiologically regulated expression profiles [166]. Hypoxia-responsive synthetic promoters represent another promising strategy for achieving spatiotemporally restricted *VEGF* expression, limiting excessive or ectopic angiogenesis under normoxic conditions while selectively enhancing gene activity within ischemic tissues [167]. Finally, cell-free scaffold-mediated gene delivery systems integrating plasmids, mRNA, or nanoparticles into biodegradable biomaterials may provide sustained local release and improved retention of therapeutic agents within ischemic muscle [168].

Collectively, these approaches may substantially enhance the efficacy and safety of future angiogenic gene therapies for CLTI. Equally important will be the implementation of more rigorous patient stratification strategies, with particular emphasis on clinically severe phenotypes, including CLTI and diabetic foot syndrome, where the unmet medical need remains greatest. The integration of these approaches may ultimately overcome the limitations inherent to early-generation clinical studies and facilitate the transition of gene therapy toward clinically meaningful therapeutic outcomes in limb ischemia.

## Figures and Tables

**Table 1:** A review of major clinical trials on gene therapy for lower limb ischemia.

Genetic Construct, Phase, Administration, Dose	Number of Participants/Age/ Sex	Stage of Ischemia, Clinical Manifestations	Primary Endpoint	Follow-Up Duration	Blinding Status	Clinical Outcomes	Adverse Effects	Country	References/Clinical Trials
			NONVIRAL METHODS						
**VM202, Plasmid HGF728+ HGF723, Phase II, 16 intramuscular injections, (i/m. inj) (total dose 8–16 mg)**	50 patients, 18–90 years M, F	CLTI with skin lesions, Fontaine stage IV.	Change from baseline in Visual Analog Scale (VAS) for pain.	12 months	Randomized, double-blind, placebo-controlled	Higher complete ulcer healing with high dose; ≥50% ulcer area reduction in both high- and low-dose groups; Improved TcPO_2_ at 12 months with high dose.	No serious gene-therapy-related adverse events.	USA, Republic of Korea	[112]; ***NCT01064440***
**VM202 (ENGENSIS; plasmid HGF728/HGF723), Phase I/II, 2 sets of i/m. inj. in 2 weeks; 3 cohorts with dose 4; 8; 16 mg. (total dose 4–16 mg)**	28 patients, 58.8 ± 8.8 years M, F	CLTI with ischemic rest pain and/or ulcers, Fontaine stage III–IV	Safety and reduction in ischemic rest pain	6 months	Randomized, double-blind, placebo-controlled	Improvement in ischemic pain	No serious treatment-related adverse events observed	USA	[113]; **n.a.**
**HGF plasmid (HGF-0205), Phase II, 3 sets of 8 i/m. inj every 2 weeks; (total dose 4 mg)**	27 patients, M, F	CLTI with rest pain and with skin lesions, Fontaine stage III–IV	Wound healing; Reduction in major amputation; Change from baseline in Visual Analog Scale (VAS) for pain. TBI (Toe brachial index) improvement.	6 months	Randomized, double-blind, placebo-controlled	No significant difference in wound healing; No significant difference in major amputation; TBI significantly improved; Pain reduction significantly improved.	No serious gene therapy-related adverse events; treatment generally well tolerated	USA	[110]; **n.a.**
**HGF Plasmid (Kollategene), Phase III, 2 cycles, each consisting of 2 courses of i/m. inj Cycle 1: Day 0 and Month 3; Cycle 2: Month 9 and Month 12. Each course included 4–8 injections of 0.5 mg (total dose of 8–16 mg) **	44 patients, 40–90 years M, F	CLTI with skin lesions, Fontaine stage IV.	Reduction in major amputation or revascularization Change in ischemic rest Pain Ulcer improvement VAS improvement ABI improvement Changes in the quality of life	18 months	Randomized, double-blind, placebo-controlled	Higher improvement in pain and ulcer healing in the HGF group; Complete ulcer healing in the ulcer subgroup with sustained effect over 12–24 weeks; Significant increase in ABI and reduction in rest pain; Trend toward improved limb salvage.	Rare peripheral edema; no serious gene-therapy-related adverse events.	Canada Netherlands Sweden Belgium Hungary USA Finland Denmark Poland Italy France	[159]; ***NCT02144610***
**NV1FGF, Plasmid FGF1 (TALISMAN), Phase II, 8** ** i/m. inj ** **(total dose ~16 mg)**	125 patients, ~72 years M, F	CLTI with skin lesions, Fontaine stage IV.	Ulcer healing	12 months	Randomized, double-blind, placebo-controlled	Did not demonstrate a statistically significant improvement in ulcer healing; Reduced risk of total and major amputations; Trend toward decreased mortality, though not statistically significant.	No serious gene-therapy-related adverse events.	Belgium France Germany Italy Switzerland UK	[115]; ***NCT00368797***
**NV1FGF, Plasmid FGF1 (TAMARIS), Phase III, 4** ** i/m. inj ** **of 8 mg plasmid at 2-week intervals, (total dose ~16 mg)**	525 patients, 50–92 years M, F	CLTI with skin lesions, Fontaine stage IV.	Amputation-free survival	12 months	Randomized, double-blind, placebo-controlled	Did not demonstrate a reduction in amputation or death in patients with critical limb ischaemia; Did not show a statistically significant improvement in ulcer healing; Trend toward reduced mortality, but it was not statistically significant.	No serious gene-therapy-related adverse events.	United States Argentina Australia Austria Belarus Belgium *	[116]; ***NCT00566657***
**phVEGF165,** **Plasmid, Phase I, 2** ** i/m. inj (administration of 2 mg plasmid DNA followed by a second 2 mg dose 4 weeks later; ** **total dose 4 mg)**	9 patients 59 ± 19 years M, F	CLTI with skin lesions, Fontaine stage IV.	Safety and feasibility of intramuscular plasmid gene transfer.	6 month	Open-label; no placebo control	Improved ABI/TBI; Collateral vessel development; Ulcer healing; Pain reduction and limb salvage.	Main adverse effect was transient lower-extremity edema in 6 patients.	USA	[49]; **n.a.**
**pCMV-VEGF165 (Neovasculgen) Phase 2b/3, 2 i/m. inj of 1.2 mg of pCMV-vegf165 (total dose 2.4 mg)**	100 patients, 40–70 years M, F	CLTI, Fontaine stage IIa–III.	Pain-free walking distance (PWD), ABI improvement	24 months; additional 5-year follow-up reported separately	Randomized, controlled, open-label	Increased pain-free walking distance (PWD) at 6, 12, and 24 months; Significant improvement in ABI and blood flow velocity; No improvements observed in the control group.	No serious gene-therapy-related adverse events; treatment was generally well tolerated.	Russia	[160]; ***NCT03068585***
**pCMV-VEGF165 (Neovasculgen), 5-year follow-up after Phase IIb/III trial, 2 i/m. inj of 1.2 mg of pCMV-vegf165 (total dose 2.4 mg)**	48 patients, 40–70 years M, F	CLTI, Fontaine stage IIa–III	PWD	5 years	Open-label observational follow-up	Increased PWD; Significant improvement ABI; Significant improvement TcPO_2_.	No serious gene therapy-related adverse events; treatment was generally well tolerated.	Russia	[121]; ***NCT03068585***
** JVS-100 (SDF-1/CXCL12 plasmid), Phase 2B, i/m. inj ** **of 8 mg or 16 mg plasmid** ** (total dose 8 mg or 16 mg) **	109 patients, ~71 years with evidence of persistent impaired forefoot perfusion following recent successful revascularization M, F	CLTI with skin lesions, Fontaine stage IV.	Complete wound healing	6 months	Randomized, double-blind, placebo-controlled	No significant improvement in complete wound healing at 6 months across all groups; Increase in toe–brachial index within groups; No reduction in major adverse limb events (MALE).	No clear treatment-related safety signal but no benefit demonstrated	USA	[114]; ***NCT02544204***
**pIRES/VEGF165/HGF, randomized controlled trial, ~80** ** i/m. inj ** **(total dose** ** 4 mg) **	28 patients, 40–85 years M, F	CLTI with skin lesions, Fontaine stage IV + patients with diabetes.	ABI improvement and collateral vessel formation	3–6 months	Randomized controlled (open-label)	Significant increase in ABI from baseline; Elevated serum VEGF levels following treatment; Marked reduction in rest pain with no improvement in controls; Increased collateral vessel formation on imaging.	No serious gene-therapy-related adverse events.	Poland	[26]; **n.a.**
**pCK-HGF-X7 plasmid (NL003), Phase II, 3** ** i/m. inj ** **of 8 mg plasmid at day 0, 14, 28 (total dose 24 mg)**	197 patients ~68 years M, F	CLTI with skin lesions, Fontaine stage IV	Pain reduction Ulcer healing ABI improvement	6 months	Randomized, double-blind, placebo-controlled	Significant reduction in pain severity across all doses; Higher complete ulcer healing rate in the high-dose group; No significant changes in ABI, TcPO_2_, or TBI.	Mild injection-site reactions; no serious gene-therapy-related adverse events.	China	[161]; ***NCT04275323*, *NCT04274049***
**VIRAL METHODS**									
**RAVE** **Ad5-VEGF121** **Phase II, single i/m. inj,** **Total dose: low (4 × 10^9^ vp)-high (4 × 10^10^ vp)**	105 patients, 40–80 years M, F	CLTI, Fontaine stage IIb	Change in Peak walking time	12 weeks	Randomized, double-blind, placebo-controlled	No significant improvement in: Peak walking time; Claudication onset time; ABI; Quality-of-life scores.	Peripheral edema (dose-dependent), mild inflammatory reactions; no serious gene-therapy-related adverse events.	USA	[130]; **n.a**
**Ad2/HIF-1α/VP16** **Phase II, single i/m. inj,** **Total dose: (2 × 10^9^/10^10^ or 10^11^ vp)**	289 patients 40–80 years M, F	CLTI, Fontaine stage IIb	Change in peak walking time from baseline	12 months	Randomized, double-blind, placebo-controlled	No significant improvement in: Claudication onset time; ABI; Quality-of-life scores.	No serious gene-therapy-related adverse events.	USA UK Germany	[145]; ***NCT00117650***

* Studies are not limited to the listed countries; please refer to the provided link for the full list of participating countries.

## Data Availability

No new data were created or analyzed in this study. Data sharing is not applicable to this article.
